# 

**Published:** 1858-04

**Authors:** 


					﻿No. XI.]
APRIL.
[Vol. XIII.
BUFFALO
MEDICAL JOURNAL
AND
JUnntblij Mnriew
or
MEDICAL AND SURGICAL SCIENCE;
SANFORD B. HUNT, M. D.,
EDITOR.
AUSTIN FLINT, Jr., M. D.,
ASSOCIATE EDITOR.
BUFFALO, N. Y.:
E. R. JEWETT & CO.’S STEAM PRESS.
Commercial Advertiser Buildings.
1858.
Price 82.00 in advance or $2.50 at the end of three months.
				

